# Risk factors associated with cervical spine lesions in patients with rheumatoid arthritis: an observational study

**DOI:** 10.1186/s12891-021-04285-7

**Published:** 2021-05-03

**Authors:** Yosuke Uchino, Takayuki Higashi, Naomi Kobayashi, Tetsuhiko Inoue, Yuichi Mochida, Yutaka Inaba

**Affiliations:** 1 Department of Orthopaedic Surgery, Yokohama City University Medical Center, 4-57 Urafune- cho, Minami-ku, 232-0024 Yokohama, Japan; 2 Center for Rheumatic Diseases, Yokohama City University Medical Center, 4-57 Urafune-cho, Minami-ku, 232-0024 Yokohama, Japan; 3 Departments of Orthopaedic Surgery, Yokohama City University Graduate School of Medicine, 3-9, Fukuura, Kanazawa-ku, 236-0004 Yokohama, Japan

**Keywords:** Rheumatoid arthritis, Cervical lesions, Osteoporosis, Bone mineral density, Biological agents

## Abstract

**Background:**

Few reports have described the association between rheumatoid arthritis (RA) cervical lesions and osteoporosis, especially in patients with vertical subluxation (VS) that could be induced by the collapse of lateral masses in the upper cervical spine. Therefore, this study aimed to investigate the prevalence and risk factors for cervical lesions in patients with RA under current pharmacological treatments with biological agents, and to investigate the relationship between osteoporosis and VS development.

**Methods:**

One hundred eighty-five consecutive patients with RA who underwent both cervical plain radiography and bone mineral density (BMD) scanning were enrolled. RA cervical lesions included atlantoaxial subluxation (AAS), VS, and subaxial subluxation (SAS). We assigned patients with AAS, VS, or SAS to the cervical-lesion group, and all other patients to the non-cervical-lesion group. Radiological findings, BMD, and clinical data on RA were collected. We used multivariate logistic regression analyses to assess the risk factors for cervical lesions in patients with RA.

**Results:**

The cervical-lesion and non-cervical-lesion groups included 106 and 79 patients, respectively. There were 79 patients with AAS, 31 with VS, and 41 with SAS. The cervical-lesion group had a younger age of RA onset, longer RA disease duration, higher RA stage, and lower femoral neck BMD than the non-cervical-lesion group. Multivariate analyses showed that the risk factors for RA cervical lesions were prednisolone usage, biological agent usage, and higher RA stage. Prednisolone usage and femoral neck BMD were the risk factors for VS.

**Conclusions:**

Cervical lesions were confirmed in 57 % of the patients. Prednisolone usage, biological agent usage, and higher RA stage were significant risk factors for cervical lesions in patients with RA. The general status of osteoporosis might contribute to the development of VS.

## Background

 Rheumatoid arthritis (RA) is a type of autoimmune arthritis that causes chronic inflammatory synovitis. RA lesions also invade the spine, and cervical lesions are particularly common in RA, which results in several characteristic deformities [1]. These deformities include atlantoaxial subluxation (AAS), vertical subluxation (VS), and subaxial subluxation (SAS). They possibly lead to cervical instabilities that may cause neurologic damage and induce a fatal status due to compression of the spinal cord or brain stem [2].

The treatment of RA has changed dramatically due to recent developments in biological agents. The available biological agents are the proinflammatory cytokine therapies, such as infliximab, which act by inhibiting the expression of tumor necrosis factor α (TNFα). These drugs are effective in blocking disease activities and joint degradation [3]. The incidence of cervical spine instability is 32–42 %, which may be decreasing because of stable disease control due to the usage of biological agents [4, 5] Unfortunately, the incidence of cervical instability in patients with RA is still greater than 30 %, which represents a major health issue.

There have been several reports on RA cervical lesions [4–9]. However, few reports have described the association between RA cervical lesions and osteoporosis, especially in patients with VS that could be induced by the collapse of lateral masses in the upper cervical spine [10, 11]. Therefore, osteoporosis could affect the progression of VS; however, the association between osteoporosis and VS development remains uncertain.

The purpose of our study was to investigate the prevalence and risk factors for cervical lesions in patients with RA under current pharmacologic treatment after the approval of biological agents, and to investigate the relationship between osteoporosis and VS development.

## Methods

### Study design and population

Between 2008 and 2016, a total of 317 patients with RA who underwent cervical plain radiography were reviewed to investigate the prevalence and risk factors for cervical lesions in patients with RA under current pharmacological treatments with biological agents, and to investigate the relationship between osteoporosis and VS development. Seventeen patients who had a history of cervical surgery were excluded. Finally, 185 patients who underwent both cervical plain radiography and bone mineral density (BMD) scans were included in this study. All patients fulfilled the American Rheumatism Association RA criteria [12]. All study participants provided informed consent, and the study design was reviewed and approved by the Ethical Committee for Clinical Research at Yokohama City University Medical Center.

### Data collection

Radiological findings and BMD data were collected from the electronic medical records. Radiography was measured when the patients complained of neck pain and/or neurological abnormalities. Clinical data on RA were also obtained and included age at RA onset and RA disease duration; data on current medications, including the use of prednisolone, methotrexate (MTX), and biological agents; and previous joint surgery for RA. Additionally, the C-reactive protein level, rheumatoid factor (RF), matrix metalloproteinase-3 level, and the Disease Activity Score based on C-reactive protein (DAS28-CRP) were reviewed.

The BMD of the lumbar spine and femoral neck was measured using Hologic Horizon A dual X-ray bone densitometry (Hologic, Marlborough, MA, USA). The BMD is described as a young adult mean score. We divided the patients into two groups based on the results of their radiographic evaluation of the cervical spine: the cervical-lesion group included patients with AAS, VS, or SAS, and the non-cervical-lesion group included patients with no cervical lesions. In the cervical-lesion group, we subdivided the patients into two subgroups: the VS group that included patients with VS, and the non-VS group that included patients with no VS.

### Treatment regimen

Patients with RA received intensive therapy with disease-modifying antirheumatic drugs: methotrexate (< 16 mg/week), salazosulfapyridine (< 1.0 g/d), D-penicillamine (< 600 mg/d), and bucillamine (< 300 mg/d). Oral corticosteroids (< 20 mg/d) were also administered to relieve RA symptoms. In more severe cases, tacrolimus hydrate (< 3 mg/day), leflunomide (20 mg/day), tofacitinib citrate (10 mg/day), or biologic agents such as abatacept (500–750 mg/4 weeks), etanercept (10–25 mg/2 weeks or 25–50 mg/week), infliximab (3 mg/kg/8 weeks), tocilizumab (8 mg/kg/4 weeks), certolizumab pegol (400 mg/4 weeks), adalimumab (40 mg/2 weeks), and golimumab (50 mg/4 weeks with MTX or 100 mg/4 weeks) were administered.

### Radiographic evaluation

Lateral cervical radiographs were obtained with patients in the neutral, extension, and flexion positions using the standardized protocol (exposure time, 80 msec; distance, 150 cm; current, 250 mA; voltage, 72 kV). We measured the atlantodental interval, Ranawat value, and subluxation. AAS was defined as an atlantodental interval > 3 mm [13], VS was defined as a Ranawat value < 13 mm [14], and SAS was defined as vertebral translation > 2 mm without osteophyte formation [15].

Unilateral or bilateral hand radiographs were used to classify the severity of peripheral joint destruction based on the Steinbrocker classifications (I–IV) [16]. The radiographs were evaluated by three spine surgeons.

### Statistical analysis

The results are expressed as medians and interquartile ranges. Statistical comparisons between the groups were performed using the Student t-test, Mann−Whitney U test, chi-square test, or Fisher’s exact test, as appropriate. Multivariate logistic regression analysis was performed to assess the risk factors of cervical lesions. Variables eligible for inclusion in the multivariable analysis had *P*-values < 0.20 in the univariable analyses and were clinically and biologically plausible. To evaluate the risk factors for RA patients with high disease activity, multivariate analyses were performed for patients with RA stages III or IV separately. *P*-values < 0.05 were considered statistically significant. All statistical analyses were performed using JMP 12 (SAS Institute Inc., Cary, NC, USA).

Intraclass correlation coefficients of the intraobserver reliabilities for the parametric atlantodental interval, Renawat value and vertebral translation were 0.92, 0.81, and 0.89, respectively. Intraclass correlation coefficients of the interobserver reliabilities were 0.94, 0.87, and 0.93, respectively. Theκcoefficients of the intraobserver and interobserver reliabilities for the non-parametric RA stage were 0.74 and 0.71, respectively. All the values indicated good reproducibility.

## Results

The enrolled patients included 12 men and 173 women with a median age of 68 years. The median age at RA onset was 52 years, and the median RA disease duration was 11 years. There were 106 (57 %) patients in the cervical-lesion group and 79 (43 %) patients in the non-cervical-lesion group. There were 78 patients with AAS, 42 with SAS, and 31 with VS. The patients were administered the following biological agents: abatacept (*n* = 17), etanercept (*n* = 12), infliximab (*n* = 10), tocilizumab (*n* = 8), certolizumab pegol (*n* = 5), adalimumab (*n* = 3), and golimumab (*n* = 1) (Table 1).

**Table 1:** Patient demographics and clinical parameters

Number of patients	185
Age (years) (IQR)	68 (62−74)
Sex (n) (male:female)	12:173
Age of RA onset (years) (IQR)	52 (43−64)
RA disease duration (years) (IQR)	11 (4.8−23)
CRP level (mg/dL) (IQR)	0.3 (0.1−1.1)
RF positive (n)	53 % (98/185)
MMP3 positive (n)	55 % (102/185)
DAS28-CRP (IQR)	2.3 (1.7−3.1)
RA stage (n)	I: 10, II: 46, III: 40, IV: 89
Prednisolone (n)	49 % (90/185)
MTX (n)	63 % (116/185)
Biological agents (n)	30 % (56/185)
BMD of the lumbar spine (YAM) (IQR)	82 % (71−93)
BMD of the femoral neck (YAM) (IQR)	66 % (58−66)
Previous joint surgery (n)	22 % (40/185)
**Administered biological agents**	
Abatacept	*n* = 17
Etanercept	*n* = 12
Infliximab	*n* = 10
Tocilizumab	*n* = 8
Certolizumab pegol	*n* = 5
Adalimumab	*n* = 3
Golimumab	*n* = 1

*BMD* bone mineral density, *CRP* C-reactive protein, *DAS28-CRP* Disease Activity Score based on C-reactive protein, *MTX* methotrexate, *MMP3* matrix metalloproteinase-3, *RF* rheumatoid factor, *YAM* young adult mean, *IQR* interquartile range

In the univariate analysis comparing the cervical-lesion and non-cervical-lesion groups, the age at RA onset and BMD of the femoral neck was significantly lower in the cervical-lesion group than in the non-cervical-lesion group. The RA disease duration, ratio of RA stage III or IV, and previous joint surgery was significantly higher in the cervical-lesion group than in the non-cervical-lesion group. The usage ratios of prednisolone and biological agents were significantly higher in the cervical-lesion group than in the non-cervical-lesion group (Table 2).

**Table 2:** Comparison between the cervical and non-cervical lesion groups (univariate analysis)

	Cervical lesion group (*n* = 106)	Non-cervical lesion group (*n* = 79)	*P*-value^*^
Sex (n) (male:female)	6:100	6:73	0.60
Age (years) (IQR)	68 (62−73)	68 (62−75)	0.66
Age of onset (years)	48 (39−59)	59 (50−68)	< 0.01
RA disease duration (years) (IQR)	15 (8−26)	6 (2−13)	< 0.01
CRP level (mg/dL) (IQR)	0.4 (0.1−1.3)	0.2 (0.1−0.8)	0.06
RF positive (n)	62 % (66/106)	62 % (49/79)	0.97
MMP3 positive (n)	64 % (68/106)	67 % (53/79)	0.68
DAS28-CRP (IQR)	2.3 (1.9−3.0)	2.1 (1.6−3.1)	0.76
RA stage III or IV (n)	88 % (93/106)	46 % (36/79)	< 0.01
Prednisolone (n)	58 % (61/106)	37 % (29/79)	< 0.01
MTX (n)	60 % (64/106)	66 % (52/79)	0.45
Biological agents (n)	53 % (56/106)	19 % (15/79)	< 0.01
BMD of the lumbar spine (YAM) (IQR)	79 (70−91)	86 (78−95)	0.09
BMD of the femoral neck (YAM) (IQR)	64 (57−72)	72 (64−81)	< 0.01
Previous joint surgery (n)	41 % (43/106)	23 % (18/79)	0.01

*BMD* bone mineral density, *CRP* C-reactive protein, *DAS28-CRP* Disease Activity Score based on C-reactive protein, *MTX* methotrexate, *MMP3* matrix metalloproteinase-3, *RF* rheumatoid factor, *YAM* young adult mean, *IQR* interquartile range

^*^The *P*-value was calculated using the chi-square test, Student t-test, or Mann−Whitney U test

Multiple logistic regression analysis revealed that the ratio of RA stage III or IV and the usage of prednisolone and biological agents were significant risk factors of cervical lesions in patients with RA (Table 3). In the univariate analysis comparing the VS and non-VS groups, the age at RA onset, RA duration, ratio of RA stage III or IV, usage of prednisolone, BMD of the femoral neck, and previous joint surgery were significantly different between the groups (Table 4). Multiple logistic regression analysis of VS development showed that usage of prednisolone and BMD of the femoral neck were significant risk factors (Table 5). In RA stage III or IV patients with high disease activity, the usage of prednisolone and BMD of the femoral neck were also significant risk factors for VS development (Tables 5 and 6).

**Table 3:** Multivariate analysis of risk factors for cervical lesions

	*P*-value	Odds ratio	95 % CI
RA stage III or IV	< 0.01	4.96	2.00−12.30
Biological agents	0.03	2.46	1.07−5.65
Prednisolone	< 0.01	2.81	1.31−6.00

*CI* confidence interval, *RA* rheumatoid arthritis

**Table 4:** Comparison between the VS and non-VS groups (univariate analysis)

	VS group (*n* = 31)	Non-VS group (*n* = 154)	*P*-value^*^
Sex (male:female)	4:27	2:146	0.11
Age (years) (IQR)	67 (63−72)	68 (61−75)	0.36
Age of onset (years) (IQR)	48 (38−56)	55 (44−66)	< 0.01
RA duration (years) (IQR)	18 (13−25)	9 (4−21)	< 0.01
CRP level (mg/dL) (IQR)	0.7 (0.4−1.5)	0.3 (0.1−1.0)	< 0.01
RF positive (n)	55 % (17/31)	64 % (98/154)	0.36
MMP3 positive (n)	61 % (19/31)	66 % (102/154)	0.60
DAS28-CRP (IQR)	2.9 (2.2−3.6)	2.2 (1.7−3.0)	0.55
RA stage III or IV (n)	97 % (30/31)	64 % (99/154)	< 0.01
Prednisolone (n)	68 % (21/31)	45 % (69/154)	0.02
MTX (n)	52 % (16/31)	65 % (100/154)	0.16
Biological agents (n)	42 % (13/31)	28 % (43/154)	0.12
BMD of the lumbar spine (IQR)	79 % (74−92)	82 (71−93)	0.66
BMD of the femoral neck (IQR)	60 % (49–67)	68 (60–77)	0.04
Previous joint surgery (n)	68 % (21/31)	26 % (40/154)	< 0.01

*BMD* bone mineral density, *CRP* C-reactive protein, *DAS28-CRP* Disease Activity Score based on C-reactive protein, *MTX* methotrexate, *RA *rheumatoid arthritis, *VS* vertical subluxation, *IQR* interquartile range

^*^The *P*-value was calculated using the Fisher exact test, Mann−Whitney U test, and Student t-test, or chi-square test

**Table 5:** Multivariate analysis of the risk factors for VS development

	*P*-value	Odds ratio	95 % CI
Prednisolone	< 0.01	4.26	1.290−14.00
BMD of the femoral neck	0.02	0.89	0.852−0.936
**in RA III or IV patients**			
Prednisolone	0.04	2.92	1.07−7.98
BMD of the femoral neck	0.02	0.95	0.918−0.992

*BMD* bone mineral density, *CI* confidence interval, *RA* rheumatoid arthritis

**Table 6:** Comparison between VS and non-VS groups of RA stage III or IV patients (univariate analysis)

	VS group (*n* = 30)	Non-VS group (*n* = 99)	*P*-value^*^
Sex (n) (male:female)	4:26	2:97	0.04
Age (years) (IQR)	68 (64−72)	68 (61−73)	0.65
Age of onset (years) (IQR)	48 (39−56)	50 (40−60)	0.17
RA duration (years) (IQR)	18 (13−26)	14 (6−26)	0.10
CRP level (mg/dL) (IQR)	0.7 (0.4−1.6)	0.3 (0.1−0.8)	< 0.01
RF positive (n)	57 % (17/30)	69 % (68/99)	0.22
MMP3 (n)	63 % (19/30)	68 % (67/99)	0.66
DAS28-CRP (IQR)	2.9 (2.2−3.6)	2.1 (1.7−2.8)	0.06
Prednisolone (n)	70 % (21/30)	46 % (46/99)	0.02
MTX (n)	53 % (16/30)	66 % (65/99)	0.22
Biological agents (n)	43 % (13/30)	31 % (31/99)	0.22
BMD of the lumbar spine (IQR)	79 (74−91)	81 (70−91)	0.95
BMD of the femoral neck (IQR)	60 (49−67)	66 (59−74)	0.02
Previous joint surgery (n)	67 % (20/30)	30 % (30/99)	< 0.01

*BMD* bone mineral density, *DAS28-CRP* Disease Activity Score based on C-reactive protein, *MTX* methotrexate, *RA* rheumatoid arthritis, *VS *vertical subluxation, *RA* rheumatoid arthritis, *IQR* interquartile range

^*^The *P*-value was calculated using the Fisher exact test, Mann-Whitney U test, Student t-test, or chi-square test

## Discussion

In this study, the prevalence of patients with cervical lesions was 57 %. The ratio of RA stage III or IV and the usage of prednisolone and biological agents were identified as risk factors for cervical lesions.

The cervical spine is frequently involved in patients with RA, and neural impairment is a consequent complication. Irreversible spinal cord damage, difficulty with ambulation, respiratory dysfunction, and even sudden death may occur [2]. Once neurological deficits occur, neural impairment becomes progressive [17], and even surgical treatment cannot prevent neurological deterioration [18]. Therefore, RA cervical lesions remain a major health concern. Several studies have reported the risk factors of cervical lesions in patients with RA [6–8]. However, it has not been well investigated after the use of biological agents became more popular.

The registration of biological agents has changed the standard of care for patients with RA. The available biological agents are anti-proinflammatory cytokine therapies, such as TNFα blockers and a T-cell activation inhibitor [3].

Terashima et al.. have reported that mutilating changes at baseline, corticosteroid administration, and previous joint surgery are predictors of severe aggravation of cervical spine instabilities in RA [8]. Kaito et al.. have also reported that the disease duration and Steinbrocker stage are identified as independent risk factors for the incidence of cervical lesions [5]. Yurube et al.. identified that corticosteroid administration, Steinbrocker stage III or IV, mutilating changes at baseline, and the development of arthritis mulilans correlated with the progression to severe instability. Progressive development to mutilating changes and concomitant corticosteroid treatment are indicators for poor prognosis of the cervical spine in RA [19, 20]. In this study, the multivariate analysis showed that the ratio of RA stage III or IV and the usage of prednisolone and biological agents were risk factors for cervical lesions. Although biological agents may prevent the onset of cervical lesions, pre-existing cervical lesions cannot be reversed [9]. Patients with RA requiring cervical spine surgery tend to have higher disease activity, and in these patients, the incidence of cervical spine surgery remained unchanged despite treatment with biologic agents [21].

Several studies have shown that the prevalence of cervical lesions in patients with RA ranged from 32 to 57 % [4–8]. Fujiwara et al.. prospectively examined 173 patients to clarify the development and progression of cervical spinal involvement in RA. The incidences of cervical lesions were 43 % at 12.3 years of RA and 57 % at 16.5 years of RA. As follow-up proceeded, more cases of cervical lesions became apparent, indicating that cervical lesions are progressive [6]. In Japan, biological agents were approved in 2003. This might have had an influence on stabilizing RA cervical lesions. In fact, Takahashi et al.. have reported that 42 % of 220 patients from 2010 to 2011 had cervical spine instability. The prevalence has decreased since the approval of biological agents. However, there were no effects of MTX and biological agents on cervical instability [4]. Morita et al.. compared the 1999 and 2015 surveys and reported that the incidences decreased by 50 % for AAS and 75 % for VS by modern therapeutic strategies [22]. Kaito et al.. have reported that the incidence of patients with RA onset after 2000 with any cervical lesions was 31.8 %. Biological agents effectively prevented the emergence of new cervical lesions; however, they could not prevent the progression of pre-existing cervical lesions [5, 9].

Our study indicated that the prevalence of patients with cervical lesions was 57 %. There were 78 patients with AAS, 42 with SAS, and 31 with VS. This prevalence is higher than that in recent studies in this era of biological agents [4, 5]. This might have been due to the mean RA disease duration in our study, which was 14.3 years compared to the 8.5 and 11.1 years reported in previous studies [4, 5]. Additionally, a large proportion of patients with more advanced diseases might have been treated with biological agents.

Few reports have described the association between RA cervical lesions and osteoporosis. Neva et al.. have reported that the severity of atlantoaxial disorders positively correlated with the grade of destruction in evaluated joints. Furthermore, patients with atlantoaxial disorders presented with decreased BMD of the femoral neck [10]. Han et al.. observed that osteoporosis is an independent predictive factor for higher AAS occurrence in patients with a lower body mass index (BMI). The effect of a lower BMD, which is related to higher disease activity and peripheral bone erosions, on increased atlantodental interval and higher AAS development, may be synergistic in patients with RA with a lower BMI. Osteoporotic conditions may lead to atlantoaxial ligament laxity. These conditions may be aggravated in RA patients with a lower BMI [23].

VS usually occurs after AAS. VS is considered to be a serious condition in patients with RA as it can be associated with a poor survival rate and sudden death [2]. Dokai et al.. hypothesized that osteoporosis could affect the progression of VS. Their results indicated that VS could be induced by collapse of the lateral masses in the upper cervical spine. The risk factors for VS development were age, RA symptom duration, and BMD (lumbar). However, they could not show a statistically significant relationship between osteoporosis and VS development [11]. In our study, multiple logistic regression analysis revealed that the usage of prednisolone and BMD of the femoral neck were significant risk factors of development of VS. However, the difference in BMD of the lumbar spine did not show any statistical significance. This discrepancy might be due to osteophytes and endplate erosions that have been described to affect the measurement of BMD at the lumbar spine [10, 24]. Nevertheless, our results indicated that the general status of osteoporosis could contribute to the development of VS. Osteoporosis may affect the complex upper cervical structure of the bones and ligaments. However, the underlying mechanism remains unclear. Thus, further studies are needed to confirm our findings.

An appropriate treatment for BMD deficiency may prevent the development of VS. Tanaka et al.. have reported that denosumab could prevent the progression of bone erosion in the early stage of RA and play a useful role in anti-osteoporotic therapy. However, denosumab has no effect on joint inflammation or cartilage destruction in RA [25]. The complex osteoimmunological network in patients with RA suggests that the powerful anti-inflammatory activity of biological agents beyond the control of the disease is likely to reduce osteoporosis and fracture risk [26]. Therefore, a combination of biological agents and denosumab may be effective in preventing VS. Further studies are required to establish these treatments.

There are several limitations to this study. First, in this cross-sectional study, we reviewed patients with varying degrees of spinal instability, and we did not consider the duration of RA treatment and medication dosages. This might have led to a selection bias, which might have resulted in the use of biological agents being identified as a risk factor for RA cervical lesions. Second, we did not assess the data on clinical and neurological symptoms. Only patients with RA who complained of symptoms of cervical lesions were included in this study. It is possible that only more frail patients underwent a bone scan, as opposed to all patients with RA, to limit selection bias towards patients with lower BMD. Third, this was a cross-sectional study; consequently, disease activity was only reflected over a certain period. A longitudinal evaluation was not performed. Fourth, we did not perform a power analysis for the sample size. We chose the young adult mean as a variable in the osteoporosis index instead of BMD, as we were unable to find a suitable previous study that had compared RA cervical lesions and BMD.

## Conclusions

In conclusion, the prevalence of patients with cervical lesions was 57 %. The incidence of RA remains a major health issue.

Age at RA onset and BMD of the femoral neck were significantly lower in the cervical-lesion group than in the non-cervical-lesion group. The RA disease duration, higher RA stage, and previous joint surgery were significantly higher in the cervical-lesion group than in the non-cervical-lesion group. A higher RA stage and the usage of prednisolone and biological agents were significant risk factors for cervical lesions in patients with RA. The usage of prednisolone and BMD of the femoral neck were risk factors for VS development. Finally, the general status of osteoporosis could contribute to the development of VS.

## Data Availability

The datasets used and analyzed during the current study available from the corresponding author on reasonable request.

## Abbreviations

AAS: Atlantoaxial subluxation
BMD: Bone mineral density
RA: Rheumatoid arthritis
SAS: Subaxial subluxation
TNFα: Tumor necrosis factor α
VS: Vertical subluxation
